# Supplementary material for: Learning curve and surgical outcome of robotic assisted colorectal surgery with ERAS program
Source: Sci Rep. 2022 Nov 29;12:20566. doi: 10.1038/s41598-022-24665-w (PMC9709162; doi:10.1038/s41598-022-24665-w)
Supplement: Supplementary file 1 — Supplementary Information 1. [file 41598_2022_24665_MOESM1_ESM.zip › LSC Raw data-Ñ[▒K/113/O_ñw╝╨░O▒Kñσ.pdf]

正式摘要(電子簽章：000000208565753)

W55 / W55

臺中榮民總醫院

Operation Note

[redacted]

- 手術日期：2021/01/21
- 手術醫師：VS 蔣鋒帆，CR 陳映菁，CR 江世偉，R 陳凱祥
- Anesthesia : GE
- Position : Lithotomy
- Aseptic agents : Soap , Betadine Alcohol , Betadine Aqua

Diagnosis

- Pre-operative diagnosis : Malignant tumor of D-colon
- Post-operative diagnosis : Malignant tumor of T-colon

Surgical Indication

- curative treatment

Operation

- 1.Laparoscopic converted to conventional subtotal colectomy (due to severe adhesion) with end to end anastomosis, Endo-GIA 60/4.3mm, CDH 29mm, Re-inforcement with silk: nil, air leak test: yes, no leakage.
  2. Vessels ligation of right colic, middle colic artery, left colic and IMA
  3. Cut margin: proximal 25cm, distal 25cm
  4. One 12 Fr.Penrose drain placed in the pelvis

Finding

- 1. An ulcerative tumor, Borrmann's type III, about 3cm x 3cm in size, at middle transverse colon, which penetrated through muscular layer but didn't invaded to serosa layer. cT3-4N0M0
  2. A-colon diverticulum
  3. No enlarged LNs was noted along the mesocolon or intermediate vessel
  4. There was no visible liver metastasis and no visible peritoneal seeding and carcinomatosis.
  5. No synchronous tumor or polyps in the resected colon.

Procedure

- General endotracheal anesthesia was induced and then the patient was placed in modified Trendelenburg lithotomy position. Preoperative antibiotics were given. A Foley catheter and nasogastric tube were placed. The abdomen was prepped and draped in standard sterile fashion. A small vertical incision was made above the umbilicus and a 12mm trochar was inserted into the peritoneal cavity by open method. Peumoperitoneum was established with CO2 inflation. Laparoscopy was inserted and then another 12mm trochars were inserted over RLQ. Three 5mm trochars were inserted over RUQ, LUQ and LLQ of abdomen, respectively. The operative findings were mentioned as above. Severe adhesion to peritoneum was lysised, however, operation was difficult to continue under severe adhesion, then we convert laparoscopic to conventional operation. Para-umbilical wound was extended to midline incision. The small bowel was wraped with moisured pad and left it outside of abdomen. The sigmoid colon was then held by operator and the peritoneum overlying the mesentery was then scored with electrocautery. The inferior mesentery artery was identified, ligated near its origin from the aorta/below the left colic artery with 3-0 tie, and transected, so was inferior mesentery vein. Tumor was found at middle T-colon. It is then decided that subtotal colectomy should be performed. The right colon was mobilized by incising the peritoneal attachment from the cecum to the hepatic flexure. The greater omentum was then taken from the transverse

- colon up to the splenic flexure. The lateral peritoneal attachment of the left colon was then incised from the splenic flexure to the sigmoid colon. The mesentery was scored and divided. Mesenteric vessels were secured with ties and suture ligatures of 2-0 silk. The plane anterior to the aorta was entered and colon, mesenteric fat were mobilized into the lower S-colon. The mesocolon was then mobilized using sharp and blunt dissection. The RS colon was carefully elevated and then a endo GIA-60-4.3mm was used to divide the RS colon. A point of transection was selected at terminal ileum and the bowel was divided. It was determined that a 29 mm circular stapler could be accommodated. A purse-string suture of 3-0 Prolene was placed, the anvil was inserted, and then the purse-string suture was tied. The anal canal was irrigated with distil water and then the circular stapler was introduced through the anus and pierced through the mid-portion of the stapler line. The proximal bowel with anvil in place was engaged with the circular stapler and the stapler was then fired. Two complete donuts were obtained and submitted to pathology. The wound was closed with interrupted sutures of No.1 Vicryl. The fascia of the one 12 mm trochar wound was closed with 1-0 Dexon. Skin was closed with 3-0 nylon. The patient tolerated the procedure well and was brought to the POR in stable condition.
- Estimated blood loss: minimum
- 器械數量: 正確
- 紗布數量: 正確

Image

- PAINT
    - colon & rectum

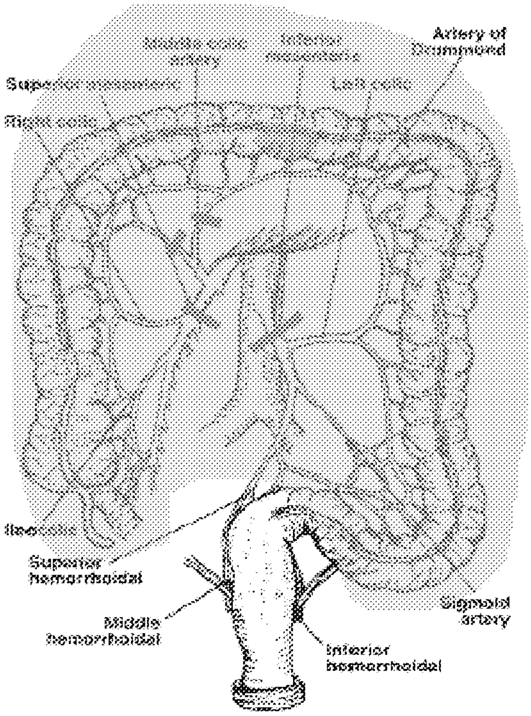

    - abdomen

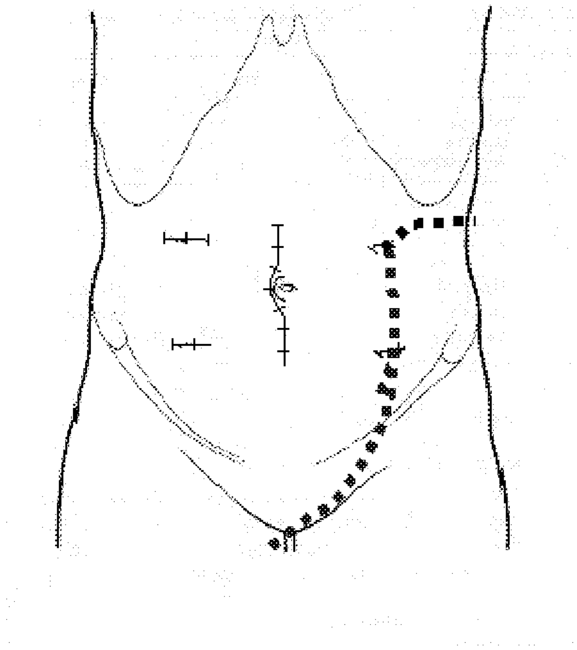

主任 2062F蔣鋒帆 / 醫師 2303K陳映菁

正式摘要(電子簽章：000000208565753)

W55 / W55

doctorRank:B
